# An Update on the Sense of Taste in Chickens: A Better Developed System than Previously Appreciated

**DOI:** 10.4172/2155-9600.1000686

**Published:** 2018-03-31

**Authors:** Hong-Xiang Liu, Prasangi Rajapaksha, Zhonghou Wang, Naomi E Kramer, Brett J Marshall

**Affiliations:** Bioscience Center, Department of Animal and Dairy Science, College of Agricultural and Environmental Sciences, University of Georgia, USA

**Keywords:** Chicken, Taste, Poultry, Feed, Nutrition

## Abstract

Taste is important in guiding nutritive choices and motivating food intake. The sensory organs for taste are the taste buds, that transduce gustatory stimuli into neural signals. It has been reported that chickens have a low taste bud number and thus low taste acuity. However, more recent studies indicate that chickens have a well-developed taste system and the reported number and distribution of taste buds may have been significantly underestimated. Chickens, as a well-established animal model for research, are also the major species of animals in the poultry industry. Thus, a clear understanding of taste organ formation and the effects of taste sensation on nutrition and feeding practices is important for improving livestock production strategies. In this review, we provide an update on recent findings in chicken taste buds and taste sensation indicating that the chicken taste organ is better developed than previously thought and can serve as an ideal system for multidisciplinary studies including organogenesis, regenerative medicine, feeding and nutritional choices.

## Introduction

Taste sensation is conserved in vertebrate animals – most species have a well-developed taste system comprised of taste sensory organs, the innervating nerves, and the central nervous system. In this review we will focus on the sense of taste in chickens, including their sensory organs and behavioral responses.

Recent studies have advanced our understanding of basic information concerning chicken taste bud number, distribution, structure, and development. Using molecular markers to label chicken taste buds in the oral epithelial sheet, many more taste buds have been observed. This indicates that chickens have a better developed taste system and thus a larger impact of taste on their feeding behavior than previously appreciated.

## Sensory Organs for Taste in Chickens

The sensory organs for taste are taste buds, which detect different types of tastants and transduce gustatory stimuli into neural signals conveyed to the brain for taste perception. Among different species, taste bud distribution varies. For example, mammalian taste buds are primarily located in the tongue, though they are also observed in the soft palate, epiglottis, pharynx, larynx, uvula [1-4]. In contrast, the avian taste organ system is a prominent example of a non-lingual taste system. Chicken taste buds differ from those of mammals in many respects.

### Taste bud number, distribution, and structure

Initially, it was reported that chickens do not have taste buds [5] and later about 70 taste buds were found in the oral cavity [6]. This number is low compared to mammals, e.g., rats (∼1000 taste buds), humans (∼10,000 taste buds) [1] and cattle, which have about 15,000-20,000 taste buds [7]. Further studies demonstrated that chickens have a higher number of taste buds varying in number from 240-360 on average according to the breed, e.g., broilers have more taste buds compared to the layer-type [8-10]. In addition, our recent studies have demonstrated that the number of taste buds varies among lines in the same type (broiler-type) of chickens at P3, i.e., female line males have more taste buds in the base of the oral cavity than females and male line males [11].

Chicken taste buds are present in both the anterior and posterior regions of the oral cavity, primarily distributed in three regions of oral epithelium– the palate (∼69%) (anterior maxillary gland opening region, middle palatine papilla region, and posterior region), the base of the oral cavity (anterior mandibular gland region) (∼29%), and posterior ventrolateral regions of the keratinized anterior tongue and posterior region of the tongue (region posterior to the lingual spine) (∼2%) [8,9]. In the oral epithelium, taste buds are mainly located near salivary gland openings (>20 μm in diameter) [8,11]. The two clusters of taste buds in the anterior maxillary gland opening region of the palate are large and dense which may be important for immediate detection as soon as the feed enters the oral cavity. The lower number of lingual taste buds in chickens suggests that the tongue is not the primary organ for taste in chickens; rather it primarily facilitates food processing.

Chicken taste buds, comprised of a cluster of specified fusiform cells, are ovoid (“egg” shaped), which is in contrast to mammals whose taste buds are onion-like/bud-shaped. Using 2-photon microscopy in the oral epithelial sheet immunostained with molecular markers, large tube-like taste buds were also seen in the posterior region of the palate [11]. Unlike mammals, chickens do not have specialized structures like lingual taste papillae (i.e., fungiform, foliate, circumvallate) to host taste buds. The taste buds are embedded in the epithelium, and grouped in clusters that surround the salivary gland openings in a rosette pattern [8,11]. Regarding the distribution of taste buds, we found that taste buds in the base of oral cavity extend to the lateral edge [11], indicating a broader distribution than previously observed.

The previous data on the number and distribution of taste buds have been obtained from the observation of taste pores (2-10 μm in diameter) with scanning electron microscopy [8]. More recently, we used molecular markers, α-Gustducin and Vimentin, to label chicken taste buds in the peeled epithelial sheet and found many more taste buds than previously reported [11]. The numbers vary by gender even in the same breed (COBB 500) – female-line male chickens have more taste buds than females and male-line males. The broiler-type female-line male chickens have up to 500 taste buds in the palate and ∼260 in the base of oral cavity at P3, which is much higher than the previously reported number with scanning electron microscopy (218 in palate and 91 in the base of oral cavity) [8]. It has been reported that the ratio of salivary gland openings to taste buds in chickens is around 1:2.5, a ratio which does not change with age [9]. However, using molecular markers to label taste buds, larger clusters are observed in the base of the oral cavity, e.g., 4 buds per cluster on average (up to 14) in males and 3 buds in females on average (up to 9) [11]. The high number of chicken taste buds implicates a better-developed taste organ system and potentially larger than previously appreciated impact of taste on feeding behaviors in birds.

Previous studies have shown that the total number of taste buds is similar in young and adult chickens [12]. However, a more detailed analysis showed that the numbers of taste buds and clusters change with age from P0-P8. For instance, taste bud number in broiler-type, female line males increases after hatch, peaks at P3, and then decreases at later stages. The continuing development of taste buds in chicks after P0 provides a time window when taste bud formation, and therefore taste sensing, can be modified in early hatched chickens [11].

### Taste bud cell types

Similar to mammals, the chicken taste bud cell population is heterogeneous histologically [8,13]. In mammals, there are four types of taste bud cells, type I cells (dark), type II cells (light) that are considered to be “receptor cells”, type III (intermediate) and type IV (basal) cells. In chickens, different taste bud cell types have been identified based on ultra-structural studies using transmission electron microscopy.

At least four taste bud cell types have been classified based on appearance, including basal cells, dark cells, light cells, and flattened/ intermediate cells [14]. The dark cells, the most abundant cell type in chicken taste buds, have cytoplasmic extensions (similar to microvilli in mammalian taste bud cells) and their main function is regarded as support of the taste bud [15]. In addition, dark cells have dense cytoplasm with scattered chromatin and fewer vesicles. The light cells, similar to type II taste bud cells in mammals, have less dense cytoplasm but more vesicles compared to dark cells. The intermediate cells have certain degrees of characteristics of both light and dark cells [14]. Basal cells, as the name indicates, are located in the basal region of the taste buds, have an irregular shaped nucleus, dense cytoplasm and are darker than the other cell types [14].

Specific markers for the three types of differentiated taste bud cells in rodents have been well-characterized, e.g., NTPDase II for type I [16], α-Gustducin for type II [17,18], SNAP25 for type III [19]. However, there are not established molecular markers to identify the specific taste cell types in chickens, although molecular markers (Vimentin and α-Gustducin) are available to label taste buds [11,20-22].

### Development and maintenance of taste buds

#### Chicken taste bud development

Chicken beaks and tongue, the regions where the oral tissues hosting taste buds are located, develop by embryonic day (E) 8, and taste buds emerge at E17 (Hamburger Hamilton stage 43) as spherical cluster of cells in the base of the epithelium. From E17-18, the cell clusters further develop and increase in number, though without a pore penetrating the surface of the epithelium. At E19, taste bud cells elongate and form an ovoid structure with a narrow and shallow taste pore penetrating the surface of the epithelium [12]. Compared to rodents, chicken taste buds develop and mature early, i.e., before hatch [23]. In rodents, taste bud development and maturation occur after birth, and taste bud maturation is completed 2-3 weeks after birth [24].

At E19, the number of taste buds peaks and has been reported to remain consistent after hatch in most chickens [12]. It has been assumed that taste bud number remained relatively static in young chicks versus adults, with no age-related effects on taste bud development [25]. However, as discussed earlier, in broiler-type Cobb 500 chickens, the female-line males continue to develop taste buds after hatch, peaking at P3, and then decreasing to a stable level [11].

#### Chicken taste bud maintenance

Taste buds reside in the epithelium and contain cells which possess epithelial features – they have a short life span and undergo continuous turnover. It has been suggested that the cell turnover rate depends on the species, age, and location of the taste buds [26,27]. In chickens, the average life span of taste bud cells is shorter compared to other vertebrates that have been reported so far. The life span of chicken taste bud cells in the base of the oral cavity is 3-4 days on average [26,27], in contrast to 7-14 days in mammals [26,28,29]. The high turnover rate of taste bud cells requires a rigorous progenitor/stem cell niche in order to maintain proper taste function. Type IV/basal cells are regarded as one of these stem cell niches. In addition, the “edge/perigemmal” cells immediately surrounding taste buds are highly proliferative which is implicit for their function in taste bud cell renewal.

Our recent studies have demonstrated a population of proliferating cells within chicken taste buds that are primarily not labeled by other known chicken taste cell markers (i.e., Vimentin and α-Gustducin), implying that these are a distinct and undifferentiated population of taste cells. This is unique because in rodents, taste bud cells are largely post-mitotic – their proliferating cells existing chiefly in the surrounding epithelium [30] and potentially the connective tissue [31]. The unique localization of proliferating cells in chicken taste buds, lining its basal layer, suggests the possibility that the taste buds are predominantly independent structures with “built-in” progenitors to meet the needs of their large size and rapid turnover. However, proliferating cells are also located in other tissue compartments such as the epithelium and connective tissue, so lineage tracing must be carried out to confirm this conjecture.

#### Regulation of taste bud development and maintenance

Taste organs, like other epithelial appendages, require epithelial-mesenchymal interactions and involvement of multiple signaling pathways for proper formation. Sonic hedgehog (Shh) and bone morphogenetic proteins (BMP) signaling cascades have been identified in the development of the avian tongue [32], where a small population of taste buds are located. The signaling mechanisms underlying chicken taste bud development are largely unknown.

Knowledge regarding the regulation of taste organ development in rodents may give us perspectives and be beneficial for in-depth studies in chickens. In rodents, numerous molecular pathways have been identified that regulate the development of embryonic tongue, developing taste papillae, and taste buds. For example, e.g., Hedgehog [33-38], Wnt/β-catenin [39-43] TGF-β/BMP [44-47], Notch [48-50] fibroblast growth factor (FGF) [45,51,52], and Erbb [53-55].

Recently, we used RNA-Seq analysis to map the transcriptomic architecture of developing gustatory tissues in chickens [56]. Although chickens have taste buds throughout the oral cavity, including the epithelia of the palate, base of the oral cavity, and posterior tongue, analyses between the epithelia and the underlying mesenchyme of each tissue demonstrated that the epithelium of the base of the oral cavity contained the most differentially expressed genes (DEGs) related to taste, including but not limited to GNAT3 and TAS1R3. A deeper analysis of this tissue using regions of the epithelium containing (gustatory) or not containing (non-gustatory) taste buds, as well as the mesenchyme situated beneath the gustatory epithelium, revealed components of several pathways involved in organogenesis that were differentially expressed. Indeed, TGFβ/BMP, FGF, Notch, SHH, and ERbb signaling cascades were differentially expressed between gustatory epithelium and gustatory mesenchyme, implicating the regulatory roles of these tissues in taste bud development and maintenance.

In summary, chicken taste organs provide an ideal system for multidisciplinary studies, including organogenesis and regenerative medicine, considering their many features as outlined below: (1) a unique distribution pattern in the gustatory tissue of the oral cavity; (2) a much shorter lifespan (∼4 days) compared to mammals (∼10–12 days in rodents) indicating a more active progenitor cell niche and allowing for a more efficient way to study taste bud cell renewal compared to the rodent model; (3) similarity to humans in the connective tissue cell marker Vimentin being expressed in a large population of taste bud cells, suggesting a comparable mechanism underlying the contribution of connective tissue to taste buds in both organisms; (4) development of new taste buds after hatching which provides a time window to study the regulation of taste bud development; (5) other beneficial aspects of using chickens as a research model, e.g., convenience of in vivo embryo manipulation, high availability and rapid development.

## Behavioral Responses of Chickens to Taste Stimuli

Chickens respond to taste stimuli immediately after hatch, and newly hatched chicks respond to different taste stimuli and show aversion/acceptance behavior for different tastants [23,57]. Behavioral studies have identified the typical response to tastants including shaking the head, wiping the beak, and tongue/beak movements. In addition, the ability of tastants to signal to the brain has been analyzed with electroencephalogram (EEG) [58].

### Taste sensitivity of chickens

The sensitivity of taste in chickens positively correlates with the total number of taste buds, i.e., the more the taste buds, the more sensitive the bitter taste [10,59]. Broiler-type males are more sensitive to taste stimuli than layer-type males because they have more taste buds [8,9]. There has been a broad consensus that birds have a lower taste acuity compared to mammals due to their low taste bud numbers. However, the recent funding using molecular markers to label taste buds shows that birds have a well-developed taste system and a high number of taste buds relative to the volume of mouth cavity [11].

Indeed, chickens (from P0 to adult) respond to chemical stimulants (e.g., hydrochloric acid, acetic acid) even at the low concentrations (Gentle, 1972). Although taste sensitivity cannot be quantified by behavioral responses, there exists a correlation between oral response and taste sensitivity. Going forward, it will be important to establish an ideal method for evaluating the actual response to chemical stimuli.

Real-time Ca^2+^ imaging in isolated chicken taste buds has been used to investigate the responses of taste cells to bitter, salt and umami tastants, and has proved to be a functional approach for analyzing the taste senses of the chicken [60]. Moreover, a simple method developed recently for labeling chicken taste buds with molecular markers in the intact epithelial sheet of palate and base of oral cavity may provide an efficient way to determine an accurate number and overall distribution pattern of all chicken taste buds, and may facilitate studies correlating taste bud quantity with feeding behaviors in chickens [11].

### Taste quality of chickens

It has been widely accepted that there are five basic taste qualities (sweet, bitter, umami, sour, salty). The different taste receptor molecules and ion channels that are localized in the cell membrane of different types of taste bud cells are the mediating molecules for transducing different taste stimuli. Therefore, the taste receptor and ion channel gene expressions in taste bud cells are responsible for taste qualities. For example, in mammals taste quality is determined by taste receptor and channel gene expressions in different taste cell types, e.g., sweet by T1R2+T1R3 in type II, umami by T1R1+T1R3 in type II, bitter by T2Rs in type II, salt by ENaC in type I, sour by PCKD channels in type III cells [61].

Compared to mammals, chickens have fewer genes for taste receptors, e.g., lacking the taste receptor T1R2 for sweet [62,63] and the bitter taste receptor repertoire is small, consisting of only 3 members (T2R1, T2R2 and T2R7), in contrast to humans (25), cows (11), and mice (35) [64,65]. Thus, in contrast to mammals, which have five taste qualities, it is presumed that chickens are only able to detect four tastes (sour, umami, salt and bitter).

Early behavioral studies have been performed in chickens using some commonly used tastants, including sucrose, saccharine, quinine acid, sodium chloride, acetic acid, and hydrochloric acid [57]. Newly hatched chicks were able to distinguish bitter and sour tastes, exhibiting aversive responses [23,57]. Bitter stimuli (e.g., quinine chloride) activates all three bitter receptors [66] for aversive responses [57,67]. Chickens' aversive responses to quinine chloride in a dose-dependent manner are similar to mammals. In contrast, chickens do not display significant responses towards ‘sweet’ stimuli presumably due to the absence of T1R2 receptor [23,57]. Finally, chickens' umami taste is detected by the GPCR T1R family receptors (T1R1 and T1R3) [60,68,69].

In addition to the tastants described above, there are studies which indicate that chickens may have other taste qualities. For example, chicks are responsive to water while there was no significant response when using egg fluid, suggesting that water alone is a strong stimulus for birds [70]. Moreover, a recent study has demonstrated that G-protein-coupled receptor-120 mediates the response of taste cells to oleic and linoleic acid, and is regarded as one of the functional fat taste receptors in chickens [71].

Oral responses in chickens are different for unique stimuli and some stimuli are not perceived until a certain high concentration (fructose, sucrose etc.) [67]. Thus far, there has not been a standard method available to determine the taste responses. Different outcomes have been reported from individual studies which could be attributed to inconsistencies between testing methods [57,67]. Therefore, establishing a standard for studying the oral responses when subjected to unique stimuli will be important for furthering our knowledge in this area.

Although chickens have unique taste bud cell types, it is unclear whether individual cell types express specific taste receptors that contribute to specific tastes. A recent report showed that Gustducin^+^ spindle-shaped cells isolated from taste buds respond to umami and bitter taste stimuli [60], similar to Gustducin^+^ cells in mammals [18]. Gustducin is a G-protein present in type II taste bud cells in mammals, which has been reported to be expressed in sweet and bitter sensing taste cells in mice, hamsters and rats [17,72,73]. However, α-Gustducin has also been identified in a large subset of chicken taste cells [20]. The expression of α-Gustducin in chicken taste bud cells suggests a signaling mechanism that is similar to mammals. Further studies on the differentiation of individual taste cell types and the expression of different taste receptor genes specific for unique taste qualities will be significant for deepening our understanding of how taste quality is determined in chickens.

### Association of taste sensitivity and quality in chickens

In chickens, taste sensitivity is different for specific taste stimuli. For instance, chickens are more tolerant to ‘sour’ taste compared to mammals, but they are highly sensitive to ‘bitter’ taste, despite having a lower sub-type number of bitter taste receptors [74], and only two of the three sub-types responsible for bitter taste [75]. Chickens also respond strongly to umami taste stimuli that are composed of inosine-5′monophospahte and monopotassium L-glutamate, suggesting that the ‘umami’ taste is highly conserved from birds to mammals [69]. However, they are only responsive to sweet and salty taste stimuli at very high concentrations (i.e., sucrose, 0-5N) [23,57].

Taste sensitivity for specific taste qualities may be altered under certain conditions. Zinc-deficiency in chickens affects water intake and enhances responses to bitter and salt taste stimuli, in stark contrast to reports in humans and rats that zinc deficiency leads to taste loss [76,77]. Although no morphological changes in taste buds were associated with these deficiencies, feed supplemented with these minerals caused a significant increase in responses to taste stimuli [78]. Vitamin A deficiency in chickens is also reported to cause a decrease in response to taste stimuli [79], presumably because Vitamin A is important for the maintenance of integrity of the epithelium. Similarly, Vitamin A deficiency leads to a significant decrease in response to NaCl and quinine chloride stimuli in rats [80].

In summary, chickens are sensitive to taste stimuli, which is consistent to the well- developed taste system indicated by the recent studies and indicates a higher than previously thought impact on feeding behaviors. The response of birds (including chickens) to each group of taste stimuli for primary taste qualities are reviewed by Roura et al. [81]. Further behavioral studies are needed for the practical applications of taste perceptions on dietary intake in poultry industry.

## Impact of Understanding Chicken's Taste Sensation on Poultry Industry

Chickens are one of the major livestock animals used for egg production and meat. Taste buds are the sensory organs that guide nutritive choices and motivate feed intake, and thus have a direct impact on the productivity of these animals. Thorough knowledge regarding taste bud development, regulation and taste response to different stimuli helps to improve the feed efficiency, thereby increasing the productivity. Further mechanistic studies on how taste bud development and taste receptor gene expression are regulated will provide information beneficial for improving feed intake and animal performance.
